# How pupil responses track value-based decision-making during and after reinforcement learning

**DOI:** 10.1371/journal.pcbi.1006632

**Published:** 2018-11-30

**Authors:** Joanne C. Van Slooten, Sara Jahfari, Tomas Knapen, Jan Theeuwes

**Affiliations:** 1 Department of Experimental and Applied Psychology, Vrije Universiteit, Amsterdam, Noord-Holland, The Netherlands; 2 Spinoza Centre for Neuroimaging, Royal Academy of Sciences, Amsterdam, Noord-Holland, The Netherlands; 3 Department of Psychology, University of Amsterdam, Amsterdam, Noord-Holland, The Netherlands; Technische Universitat Chemnitz, GERMANY

## Abstract

Cognition can reveal itself in the pupil, as latent cognitive processes map onto specific pupil responses. For instance, the pupil dilates when we make decisions and these pupil size fluctuations reflect decision-making computations during and after a choice. Surprisingly little is known, however, about how pupil responses relate to decisions driven by the learned value of stimuli. This understanding is important, as most real-life decisions are guided by the outcomes of earlier choices. The goal of this study was to investigate which cognitive processes the pupil reflects during value-based decision-making. We used a reinforcement learning task to study pupil responses during value-based decisions and subsequent decision evaluations, employing computational modeling to quantitatively describe the underlying cognitive processes. We found that the pupil closely tracks reinforcement learning processes independently across participants and across trials. Prior to choice, the pupil dilated as a function of trial-by-trial fluctuations in value beliefs about the to-be chosen option and predicted an individual’s tendency to exploit high value options. After feedback a biphasic pupil response was observed, the amplitude of which correlated with participants’ learning rates. Furthermore, across trials, early feedback-related dilation scaled with value uncertainty, whereas later constriction scaled with signed reward prediction errors. These findings show that pupil size fluctuations can provide detailed information about the computations underlying value-based decisions and the subsequent updating of value beliefs. As these processes are affected in a host of psychiatric disorders, our results indicate that pupillometry can be used as an accessible tool to non-invasively study the processes underlying ongoing reinforcement learning in the clinic.

## Author summary

It has long been known that the pupil dilates when we decide. These pupil dilations have predominantly been linked to arousal. However, reward-related processes may trigger pupil dilations as well, as dilations have been linked to activity in the dopaminergic midbrain, a region important for reward processing and reinforcement learning. Using a learning task and a computational model to quantitatively describe the cognitive processes that drive reinforcement learning behavior, we show that the pupil closely tracks different aspects of the reinforcement learning process. Prior to making a value-based choice, pupil dilation reflected the value of the soon-to-be-chosen option. After receiving choice feedback, early dilation reflected uncertainty about the value of recent choice options, while late constriction reflected how strongly an outcome violated current value beliefs. These findings provide the novel insight that the pupil can be used to track value-based decision-making, opening up a new method for online tracking of reinforcement learning processes.

## Introduction

There is fast-growing interest to understand how the pupil, as a non-invasive proxy of neuromodulation [1], relates to cognition. Already since the 1960s, pupil dilation has been associated with the expenditure of cognitive effort [2, 3]. In more recent years, its relation to decision-making has been investigated extensively. These studies show that the pupil dilates during periods of uncertainty about incoming, task-relevant information [4–7] and after the occurrence of unexpected events that generate surprise [8–11]. Also in a gambling task, where unexpected outcomes were tied to reward, was pupil dilation associated with surprise [12]. However, in that particular study choices did not influence the outcomes of the gambles; making it impossible to learn from the outcomes of choices.

In real world encounters, people can learn from the outcomes of their choices and use this information to optimize behaviors or maximize reward. Several studies have shown that pupil dilations track such reward learning processes. During classical or Pavlovian learning [13], the pupil dilates in response to cues that predict reward [14–16] and tracks changes in reward expectations [17]. In situations that require decisions to obtain reward, baseline pupil diameter prior to a choice [18], as well as task-evoked dilations [19] predicted whether a choice would be either exploratory or exploitatory, hence, predicting the sensitivity to choose the option with the highest expected reward.

While these findings suggest that the pupil provides a promising marker of several processes and states associated with learning from reward, it remains unclear how the pupil relates to the underlying process of reinforcement learning (RL). Understanding of this relationship is important, as it could open up the possibility to continuously monitor the underlying cognitive processes that shape learning and decision-making based on reward. This knowledge would greatly increase the clinical impact of pupil size recordings, which already has shown some promising results in studies involving Parkinson’s patients [20, 21]. However, it is unclear how these processes interact with other cognitive processes such as attention, cognitive effort, and uncertainty. Here, we investigated pupil size fluctuations during value-based learning and decision-making, using a computational RL model to identify the specific influences of value-related computations on pupil size.

**Fig 1:**
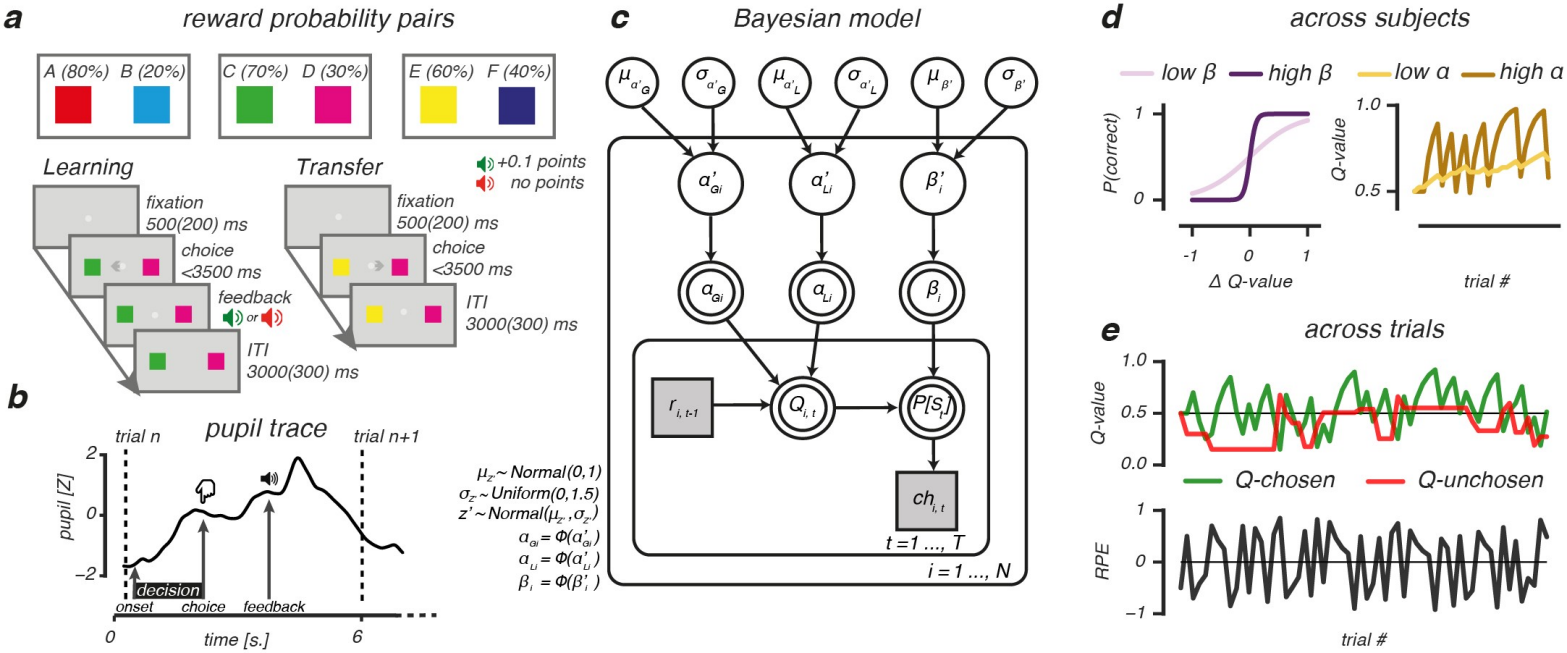
Probabilistic selection task and reinforcement learning model. (A): During learning, 3 option pairs were presented in random order. Participants had to select the more rewarding option of each pair (option A, C and E) by learning from probabilistic feedback that indicated +0.1 points reward after a “correct” choice, or no points. Choosing option A resulted in a reward in 80% of the times, whereas choosing option B resulted in a reward only in 20% of the times. Reward probability ratios were 70/30 for the CD pair and 60/40 for the EF pair, thereby increasing uncertainty about the correct option to choose. The transfer phase tested how much was learned from the probabilistic feedback. All options were randomly paired with one another, and participants selected the most rewarding option based on earlier learning. In this phase, feedback was omitted. (B): Example pupil trace for a trial in the learning phase. (C): Bayesian hierarchical model, consisting of an outer participant (*i* = 1…,*N*) and inner trial (*t* = 1…,*T*) plane. Variables of interest are depicted by circular and squared nodes, indicating continuous and discrete variables, respectively. Shaded variables are obtained from the behavioral data and used to fit the model. Double bordered variables are deterministic, as they were derived from the model fit. Arrows indicate dependencies between variables. Φ() represents the probit transform. (D): Model parameters governing value-based decision-making. Left panel: the *β*-parameter describes sensitivity to option value differences (ΔQ-value). Higher *β*-values indicate greater sensitivity to ΔQ-value and more exploitatory decisions for options with highest expected rewards. Right panel: the *α*-parameter governs value belief updating. Higher learning rates (*α*) indicate rapid, but more volatile value belief updating compared to lower learning rates. (E): Across-trial fluctuations in value beliefs (Q-values) for the chosen and unchosen option and RPEs with the EF pair as example.

We measured pupil size while thirty-four participants performed a probabilistic RL task, consisting of a separate learning and transfer phase. (Fig 1*A* and 1*B* and Methods) [22]. In the learning phase, the reliability of choice outcomes varied across three learning pairs with different reward probabilities (AB, 80:20; CD, 70:30; EF, 60:40). As participants gradually learned to choose the best option in each pair, these different reward probabilities created varying degrees of choice difficulty, uncertainty and value expectations across choices. In a subsequent transfer phase, participants were then presented with novel stimulus pair combinations (i.e., AB, CD, EF, AC, AD, AE, AF, BC, BD, BE, BF, CE, CF, DE, and DF) and asked to select the most rewarding option based on previous learning. As no choice feedback was provided in the transfer phase, it allowed us to measure how choices were guided by previously acquired reinforcement values, and how this information generalized to entirely new choice situations.

We fitted a hierarchical Bayesian version of the Q-learning RL algorithm [23] to participants’ choices in the learning phase to describe value-based choices and outcome evaluations (Fig 1*C* and Methods) [24–27]. Bayesian hierarchical parameter estimation results in more precise and stable parameter inference compared to procedures using individual-level maximum likelihood [26, 28–30], and is therefore a preferred modelling approach. The Q-learning algorithm describes value-based decision-making using two functions: a choice function and an outcome function. The choice function calculates the probability of choosing one option (Q-chosen) over the other (Q-unchosen), based on one’s sensitivity to value differences, or explore-exploit tendency (*β*; Fig 1*D*, *left panel*). The outcome function then computes the magnitude by which the reward prediction error (RPE) changes value beliefs about the chosen option, scaled by the learning rate (*α*; Fig 1*D*, *right panel*) [31]. As value beliefs are differently updated after positive and negative outcomes [32–34] via different striatal learning mechanisms [35–37], we defined separate learning rate parameters for positive (*α*_*Gain*_) and negative (*α*_*Loss*_) choice outcomes [24, 32, 33, 38].

Our computational approach allowed us to investigate the potential utility of the pupil as a proxy for value-based decision-making and value belief updating, across two levels. First, we describe participants’ choice behavior using parameters that embody core computational RL principles. These parameters provide a strong handle to investigate how inter-individual differences in value-based learning and decision-making relate to pupil responses. Second, by simulating the learning process we could investigate how pupil size depended on trial-to-trial fluctuations in underlying computational variables such as value beliefs, uncertainty and reward prediction errors. That is, our experimental paradigm allowed us to map pupil responses onto separable computational components both across participants and trials, using linear systems analysis techniques [39, 40].

## Results

### Behavioral and model performance

**Fig 2:**
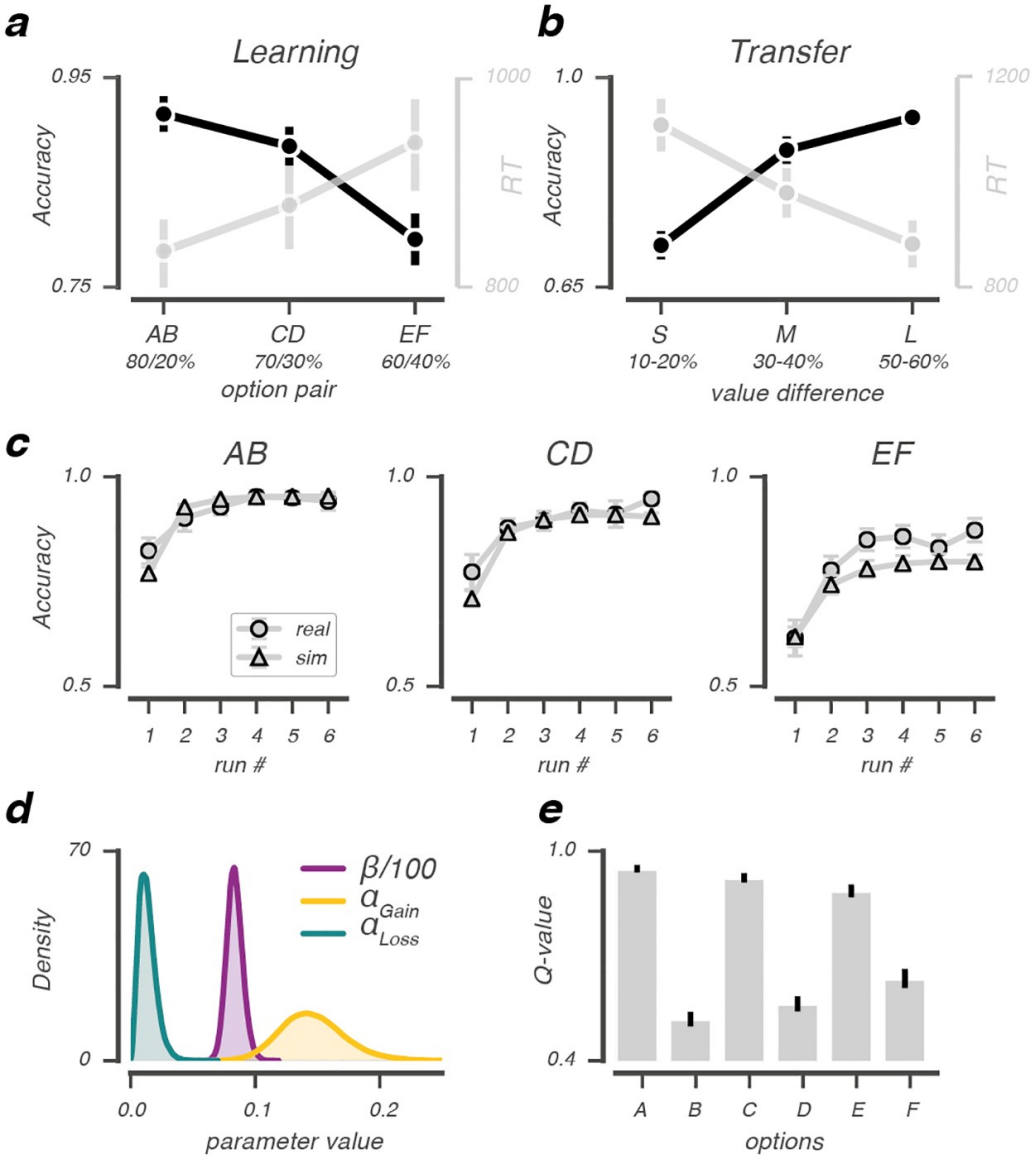
Behavioral and model performance. Average accuracy and RT across subjects (N = 34) as a function of option pairs in the learning phase (A) and option value differences (derived from the experimental reward probabilities) in the transfer phase (B) that indicated small (s), medium (m) or large (l) value differences between presented options. (C): Real and simulated choice accuracy as a function of run number in the learning phase, split by option pair. For all option pairs, simulated and real accuracy was very similar, with real EF accuracy being slightly underestimated by the model. (D): Group-level posterior distributions of the obtained parameter estimates for *β*, *α*_*Gain*_ and *α*_*Loss*_. (E): Model estimates of value beliefs for each option at the end of the learning phase. *β*/100 for visualization; error bars represent mean ± s.e.m.

Participants learned the stimulus-reward contingencies well, as they correctly learned to select the higher reward probability option in all three pairs (*P*(correct) above chance, all *P*s <.001; Fig 2*A*). Performance was best in the most reliable choice pair (AB) and decreased progressively as the feedback reliability of choice pairs decreased from CD to EF: smaller differences in the reward probability ratios increased the number of errors (*F*_(2,66)_ = 14.45, *P*<.001, $\eta_{p}^{2}=.19$) and response times (*F*_(2,66)_ = 5.5, *P* = .006, $\eta_{p}^{2}=.04$). In the transfer phase, choices were guided by the previously learned reward probabilities. Here, participants made more errors (*F*_(2,66)_ = 49.3, *P*<.001, $\eta_{p}^{2}=.53$) and were slower (*F*_(2,66)_ = 34.6, *P*<.001, $\eta_{p}^{2}=.12$) when confronted with option pairs with small value differences (Fig 2*B*), consistent with earlier studies [32, 41, 42].

The Q-learning model simulated participants’ choice behavior well (Fig 2*C*) when using the fitted learning rates (*α*_*Gain*_, *α*_*Loss*_) and explore-exploit (*β*) parameter (Fig 2*D*). In accordance with behavior, the estimated value beliefs were highest for A and lowest for B (Fig 2*E*) with differences in value beliefs being largest for AB, followed by the CD and EF pair (*F*_(2,66)_ = 20.63, *P*<.001, $\eta_{p}^{2}=.39$).

### Pupil responses predict individual differences in value-based decision-making

**Fig 3:**
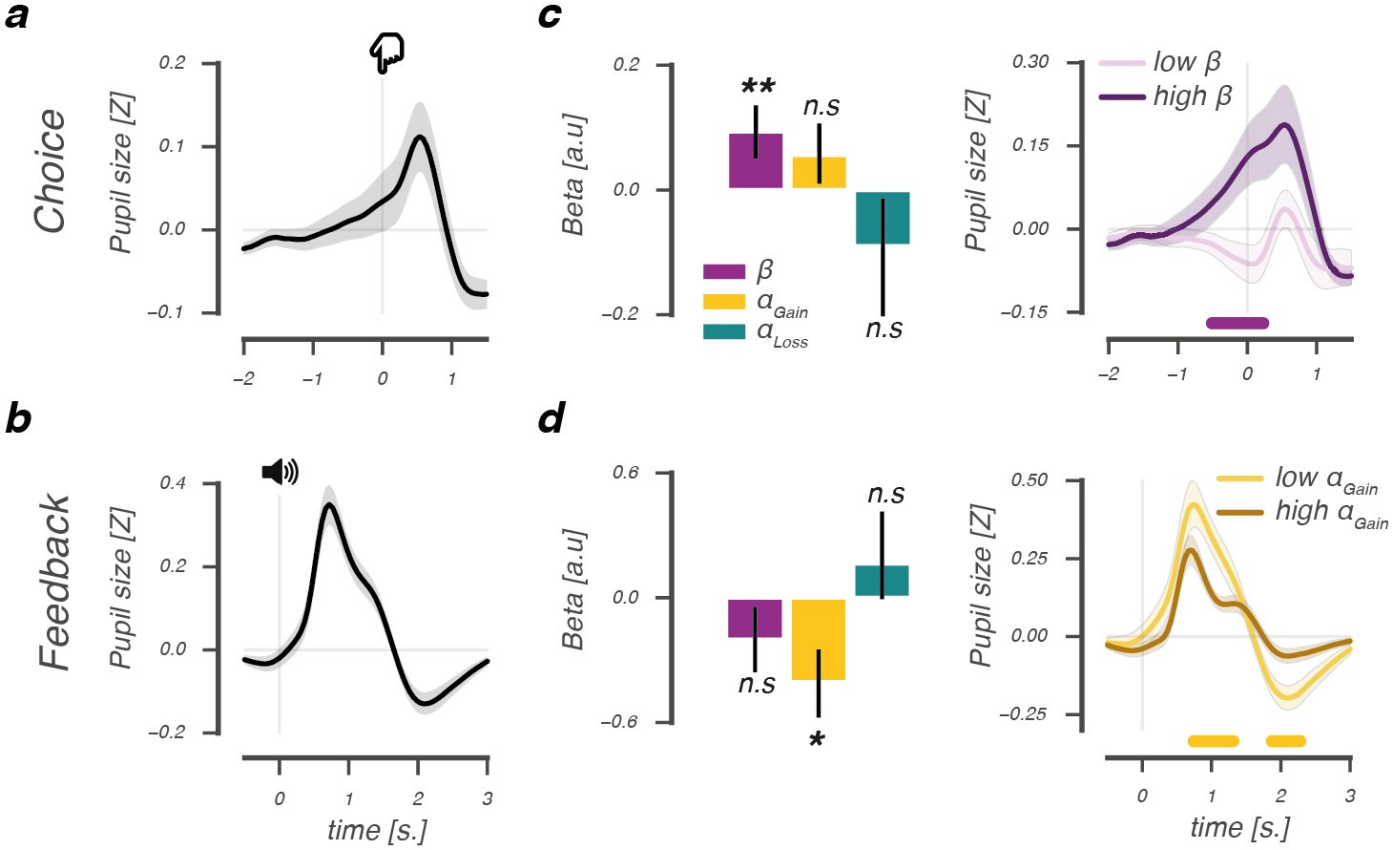
Across-subject relations between model parameters and pupil responses during choice and after feedback. Average deconvolved choice- (A) and feedback-related (B) pupil response. Regression coefficients of an across-subject GLM of the relation between derived model parameters and pupil dilation at the moment of choice (C, *upper panel*), and a scalar amplitude measure of the feedback-related pupil response (D, *upper panel*). Median split across subjects based on modulations of *β* at the time of choice (C, *lower panel*), and *α*_*Gain*_ after feedback (D, *lower panel*). Lines and (shaded) error bars of represent mean ± s.e.m of across-subject modulations (N = 34). Horizontal significance designators indicate time points where regression coefficients significantly differentiate from zero (*P*<.05), based on cluster-based permutation tests (n = 1000), **P<.01, *P<.05.

We next investigated whether the pupil was sensitive to the cognitive processes supporting value-based decisions. To do so, we first characterized the average pupil response pattern across subjects epoched around two separate moments in the trial: leading up to, and immediately after the moment of choice and around the moment of feedback. Around the moment of a choice, a biphasic pupil response was observed that was characterized by dilation starting ≈1s. prior to the moment of the behavioral report (Fig 3*A*). This upwards response reflected the unfolding decision process [5, 43] and was followed by late pupil constriction (≈1s. post-choice). After receiving choice feedback, again a biphasic pupil response was observed that was characterized by early dilation (≈1s. post-event) and late constriction (≈2s. post-event; Fig 3*B*).

Across individuals, the observed choice- and feedback-evoked pupil responses corresponded differentially to the underlying processes driving value-based decision-making. As shown in Fig 3*C*, *left panel*, pupil dilation at the moment of a choice was uniquely predicted by an individual’s sensitivity to value differences, or explore-exploit tendency (*β*; permutation test, *P* = .006; S1*A* Fig), indicating that a greater tendency to exploit high value options (high *β*) related to a stronger dilatory response (Fig 3*C*, *right panel*). Feedback-related dilation and constriction correlated inversely with an individual’s positive, but not negative, learning rate (S1*B* Fig), suggesting that this parameter selectively scaled the amplitude of the feedback-evoked pupil response. Indeed, as shown in Fig 3*D*, *left panel*, the feedback-evoked response amplitude was uniquely predicted by an individual’s positive learning rate (*α*_*Gain*_; permutation test, *P* = .017), indicating that slower updating of value beliefs after positive feedback predicted a stronger feedback-evoked response (low *α*_*Gain*_; Fig 3*D*, *right panel* and S1*D* Fig).

In sum, pupil responses evoked by choice and feedback differentially predicted the underlying processes supporting value-based decisions in the learning phase. The tendency to exploit high value options (*β*) predicted stronger pupil dilation leading up to a value-driven choice, whereas less updating of value beliefs after positive feedback (*α*_*Gain*_) predicted an amplified feedback-related response. These relations are consistent with the tenets of the Q-learning model, in which the explore-exploit parameter determines the outcome of a value-driven choice and learning rates affect how much value beliefs are updated after receiving choice feedback.

### Pupil dilation reflects the value of the upcoming choice during, but not after, reinforcement learning

We observed that across-subject variability in pupil responses was explained by model parameters that describe the underlying processes driving value-based decision-making. But do pupil responses also reflect the ongoing reinforcement learning process during value learning? In a next step, we investigated the extent to which trial-to-trial fluctuations in variables describing ongoing value-based decision-making were reflected in pupil responses.

**Fig 4:**
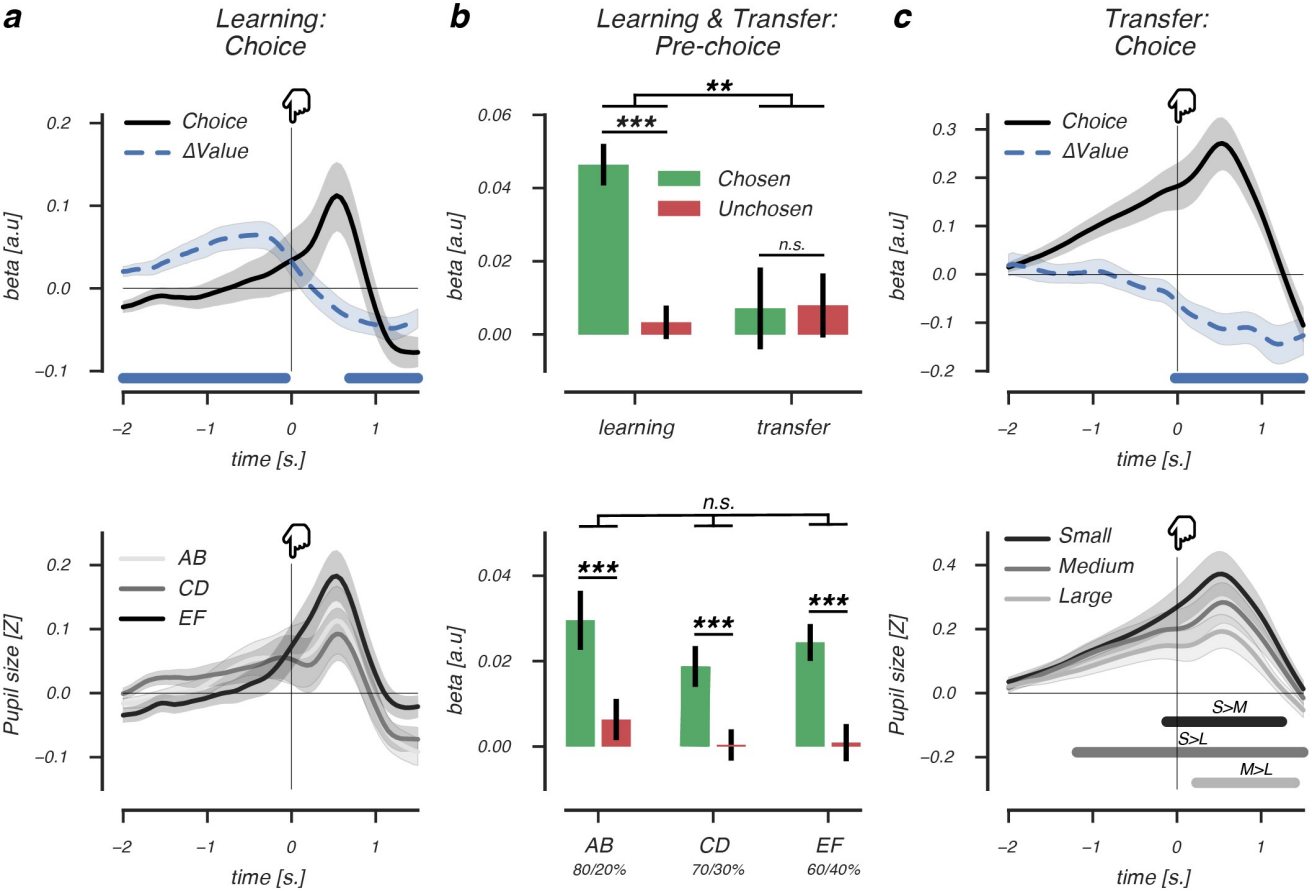
Pre-choice pupil dilation reflects the value of the upcoming choice. (A, *upper panel*): Beta coefficients accounting for choice-related pupil dilation in the learning phase. Larger value differences between options (blue dashed line) elicited larger choice-related pupil dilations (black solid line) prior to choice (at *t* = 0). After choice, this relationship reversed, as smaller value differences elicited larger post-choice pupil dilations. (A, *lower panel*): Average choice-related pupil dilation for AB, CD and EF pairs. (B,*upper panel*): Beta coefficients of chosen and unchosen value regressors accounting for pupil size fluctuations in the pre-choice decision interval of the learning (left) and transfer phase (right). (B, *lower panel*): Beta coefficients of chosen and unchosen value regressors split by learning phase pairs, showing that pre-choice pupil size is modulated by values of the to-be chosen stimulus, irrespective of uncertainty. (C, *upper panel*): Differences in learned value beliefs between the chosen and unchosen option did not modulate choice-related pupil dilation prior to choice (at *t* = 0). However, smaller value belief differences elicited stronger pupil dilation after a value-based choice. (C, *lower panel*): We defined value differences between options presented in the transfer phase using the experimentally defined reward probabilities (see also Fig 1A). Value differences ranged from 10% to 60% and were subsequently divided into three categories to describe Small (10-20%), Medium (30-40%) or Large (50-60%) value differences between presented options. Small value differences between presented options elicited largest post-choice pupil dilation, in line with findings in C, (*upper panel*), suggesting that choice conflict drove pupil size after a value-based choice. Lines and (shaded) error bars of represent mean ± s.e.m of within-subject modulations. Horizontal significance designators indicate time points where regression coefficients significantly differentiate from zero (*P*<.05), based on cluster-based permutation tests (n = 1000), *** *P* <.001, ** *P*<.01, repeated measures ANOVA.

In the learning phase, prior to reaching a value-driven choice, pupil dilation correlated positively with the value difference between options (cluster *P*<.001, 2.0s. pre-event until -0.07s. pre-event, Fig 4*A*, *upper panel*), indicating that larger value differences elicited larger pupil dilation before the choice. Specifically, the pupil dilated as a function of trial-by-trial value beliefs of the chosen, but not the unchosen option (paired *t*-test, *t*(33) = 6.98, *P*<.001; Fig 4*B*, *upper panel*), revealing that pupil dilation uniquely reflected the value belief determining the upcoming choice.

To rule out the possibility that condition differences (i.e. AB, CD, EF) instead of trial-by-trial fluctuations in chosen value beliefs explained pupil dilation prior to a choice, we estimated their independent effects on pupil size in a single regression analysis. We observed no differences between conditions in average pupil dilation prior to a choice (Fig 4*A*, *lower panel*). This also excluded the hypothesis that pre-choice pupil dilation was driven by uncertainty [5, 44] or cognitive effort [2, 3], as we did not observe significantly more dilation in the most uncertain, hence, most effortful EF pair. In all pairs, pre-choice pupil size correlated positively with chosen value (*F*_(2,66)_ = 19.76, *P*<.001, $\eta_{p}^{2}=.15$; Fig 4*B*, *lower panel*) irrespective of condition type (*F*_(2,66)_ = 1.8, *P* = .17). Thus, prior to reaching a value-driven choice, the pupil tracked subtle differences in value beliefs about the upcoming choice, while dilation did not reflect uncertainty or cognitive effort driven by condition differences.

Next, we asked whether value beliefs also modulated pre-choice pupil dilation in the subsequent transfer phase, where choices were based on previously acquired reinforcement values. In contrast to the learning phase, pupil dilation prior to a value-driven choice was not predicted by previously learned value differences between options (Fig 4*C*, *upper panel*). Indeed, a repeated measures ANOVA with the factors phase (learning, transfer) and value (chosen, unchosen) indicated that only during learning, but not during transfer, pre-choice pupil dilation was modulated by value beliefs about the upcoming choice (*F*_(1,33)_ = 6.9, *P* = .013, $\eta_{p}^{2}=.06$). This interaction effect was not explained by differences in tonic pupil size fluctuations between the experimental phases (S2 Fig), which can impact the magnitude of phasic pupil responses [1, 40]. Additionally, we investigated the observed interaction using a Bayesian repeated measured ANOVA. Compared to the null model, the model that incorporated both main factors and their interaction received most support from the data as indicated by BF = 55.4. This is regarded very strong evidence in favour of the alternative model [45]. This model also convincingly received most support from the data compared to all candidate models, as indicated by BF = 16.9. Lastly, post-hoc test that directly compared chosen and unchosen value beliefs showed that they did not differently modulate pre-choice pupil size in the transfer phase (paired *t*-test,<1, Fig 4*B*, *upper panel*). Nor did either variable’s mean correlation with pre-choice pupil dilation differ from zero(*t*<1 for chosen and unchosen value). Together, these findings provide compelling evidence that chosen and unchosen value beliefs differently modulate pupil dilation during decision formation in the learning compared to the transfer phase.

However, immediately after a value-based choice, learned value beliefs negatively predicted pupil dilation both in the learning (cluster *P* = .007, 0.68s. pre-event until 1.5s. post-event; Fig 4*A*, *upper panel*) and transfer phase (cluster *P* = .003, -0.02s. until 1.48s. post-event; Fig 4*C*, *upper panel*). Now smaller, instead of larger, value differences elicited larger post-choice pupil dilation, suggesting that the difficulty of a recent choice, or the choice conflict it generated, drove pupil size upward. Indeed, we observed a similar post-choice pupil response pattern when regressing choice conflict on the basis of the experimental reward probabilities on pupil size (Fig 4*C*, *lower panel*), indicating that post-choice pupil dilation was modulated by choice conflict, consistent with an earlier report [42].

These model-based trial-to-trial analyses show that when engaged in active reinforcement learning, pupil dilations differentially reflect value beliefs and choice conflict at different points in time. Prior to value-based choices, pupil size uniquely reflected value beliefs about the upcoming choice, where stronger dilations predicted higher value beliefs. This pattern of pre-choice value dilations was absent in the subsequent transfer phase where rewards could not be obtained, indicating that apparently similar pupil dilations prior to value-based choices can index different cognitive processes during and after reinforcement learning.

### Feedback-related pupil responses reflect value uncertainty and reward prediction errors

Only during active reinforcement learning, we observed that choice-related pupil dilation reflected value beliefs about the upcoming choice. If the pupil reliably tracked the ongoing reinforcement learning process, it should also provide information about the evaluation of a recent choice outcome. In the last step, we therefore investigated how feedback-related pupil responses covaried with the degree to which outcomes violated value beliefs about a recent choice.

**Fig 5:**
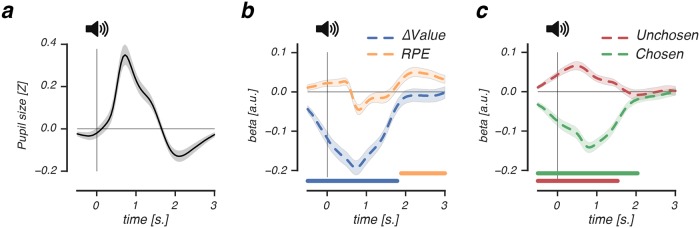
Feedback-related pupil responses reflect value uncertainty and reward prediction errors. (A, *upper panel*): Beta coefficients accounting for choice-related pupil dilation in the learning phase. Larger value differences between options (blue dashed line) elicited larger choice-related pupil dilations (black solid line) prior to choice (at *t* = 0). After choice, this relationship reversed, as smaller value differences elicited larger post-choice pupil dilations. (A, *lower panel*): Average choice-related pupil dilation for AB, CD and EF pairs. (B, *upper panel*): Beta coefficients of chosen and unchosen value regressors accounting for pupil size fluctuations in the pre-choice decision interval of the learning (left) and transfer phase (right). (B, *lower panel*): Beta coefficients of chosen and unchosen value regressors split by learning phase pairs, showing that pre-choice pupil size is modulated by values of the to-be chosen stimulus, irrespective of uncertainty. (C,*upper panel*): Learned value differences did not modulate choice-related pupil dilation prior to choice (at *t* = 0). After choice, smaller learned value differences elicited stronger pupil dilation. (C, *lower panel*): Smaller value differences between choice options elicited larger post-pupil dilation, indicating choice conflict drove pupil size. Lines and (shaded) error bars of represent mean ± s.e.m of within-subject modulations. Horizontal significance designators indicate time points where regression coefficients significantly differentiate from zero (*P*<.05), based on cluster-based permutation tests (n = 1000), *** *P* <.001, ** *P*<.01, repeated measures ANOVA.

We observed larger feedback-related pupil dilation (Fig 5*A*) after choices between options with small value differences. Specifically, early post-feedback dilation correlated negatively with differences in value beliefs of recently presented options (cluster *P*<.001, -1.5s. pre-event until 1.78s. post-event; Fig 5*B*). We furthermore verified that these feedback-related dilations were not driven by feedback valence (S3*A* and S3*B* Fig). In contrast to dilation in the choice interval, dilation in the feedback interval was explained by fluctuations in trial-by-trial value beliefs of both the chosen and the unchosen options, in opposite directions (Fig 5*C*). Thus, lower beliefs about the chosen and higher beliefs about the alternative option both increased dilation, indicating that uncertainty about the value of a recent choice modulated feedback-related dilation. In support of this, trial-by-trial chosen and unchosen value beliefs explained feedback-related dilation already prior to receiving feedback, which suggested that uncertainty about the outcome of a value-based decision drove pupil size. Lastly, outcomes that violated value beliefs did not elicit larger feedback-related dilations (S3 Fig), excluding the hypothesis that these modulations of the feedback response reflected surprise.

Importantly, whereas value beliefs about a recent choice affected early dilation, the degree to which outcomes violated those beliefs modulated late feedback-related pupil constriction. As shown in Fig 5*B*, signed RPEs correlated positively with late feedback-related pupil constriction ≈2s. after receiving feedback (cluster *P*<.001, 1.8s. until 3.0s. post-event). This correlation indicated that worse-than-expected outcomes (-RPEs) elicited stronger pupil constriction compared to better-than-expected outcomes (+RPEs).

To summarize, we observed a biphasic feedback-related pupil response that tracked the evaluation of a recent value-based choice. Early pupil dilation was modulated by uncertainty about the value of options, as choices between similarly valued options increased dilation the most. Late pupil constriction was modulated by the violation of current value beliefs, as worse-than-expected outcomes elicited stronger pupil constriction compared to better-than-expected ones.

## Discussion

The present results provide the novel insight that the pupil reliably tracks the underlying cognitive processes of learning and decision-making based on reward. When engaged in active reinforcement learning, but not when choice value was already internalized, the pupil showed two distinct response patterns. Prior to reaching a value-driven choice, pupil dilations scaled with trial-by-trial value beliefs about the upcoming choice and were diagnostic for an individual’s sensitivity to choose the option with the highest expected outcome. Feedback about the choice subsequently evoked a biphasic evaluation response. Early pupil dilation scaled with uncertainty about the value of recent choice options, whereas subsequent pupil constriction scaled with the violation of current choice value beliefs, or signed reward prediction errors.

Earlier studies have shown that pupil dilations can reflect variables or states related to reward learning [14–16, 20, 21]. Our results extend these findings by showing that pupil responses reliably track choice value computations during both decision formation and decision evaluation. The specificity of our findings outlines how the pupil can be used to index the ongoing reinforcement learning process. These results could greatly increase the clinical impact of pupil size recordings, as our findings suggest that pupil responses can be used to monitor the (affected) ongoing reinforcement learning process.

Specifically, single-trial fluctuations in pupil dilation prior to choice signaled the value of the to-be-chosen option, but not the alternative one. This indicates that during decision formation, pupil dilation specifically reflected the value that was driving the choice. Could these value-driven dilations reflect the effects of cognitive effort [2, 3], or uncertainty [4, 5, 8] that are known to affect pupil size? While effects of cognitive effort are typically studied with tasks in the domain of cognitive control (e.g. [46–49]), it is generally found that pupil dilation increases with increasing task demands. Presumably, dilations reflect the effort exerted in reaction to difficult or demanding situations [50]. In our study, this would translate to the following hypothesis: the most difficult choice condition (i.e. the most unreliably reinforced stimulus pair EF) should elicit greater pupil dilation. Our findings do not agree with this hypothesis, as choices in the difficult EF pair were not preceded by stronger pupil dilation. Neither can our findings be explained by effects of uncertainty about a value-based choice, as higher value beliefs, indicating more certainty about choice value, elicited greater dilation. What our findings indicate is that higher value beliefs about the upcoming choice led to stronger reward expectations [20, 21], or lower risk assessment [12] that increased pupil dilation prior to a value-based choice.

Furthermore, an individual’s sensitivity to value differences between presented options, as quantified by the *β* parameter of our model, predicted the amount of pupil dilation exactly at the moment of a value-based choice. Individuals that were sensitive to small value differences showed stronger choice-locked pupil dilation and made more exploitatory choices, which also led to better task performance [25]. Optimal task performance [51, 52] as well as the tendency to exploit in dynamic environments [19, 53] have previously been associated with increased choice-related pupil dilation. The observed relationship could therefore reflect either one of these processes, as choosing a high value option can result from accurate option value representations, or from the general tendency to favor exploitation over exploration [54]. Future studies that measure pupil size during value-based decision-making in a reversal learning paradigm may be able to disentangle these two alternative explanations, as optimal task performance would then depend on changing decision strategies over time.

After receiving feedback, a biphasic feedback-related pupil response tracked two different evaluation processes associated with the outcome of a choice. First, early dilation was scaled by the uncertainty associated with the outcome of a value-based choice. This was further evidenced by the observation that choices between closely valued options triggered uncertainty-related pupil dilation already prior to the moment of feedback. This suggests pupil dilation reflected value uncertainty when participants anticipated the outcome of a choice. While our study is the first to relate feedback-related pupil dilation directly to uncertainty about internal choice value beliefs, these findings are consistent with studies that relate pupil dilation to perceptual decision uncertainty, driven by observer’s internal noise [6, 7].

Second, late pupil constriction was explained by signed reward prediction errors, reflecting how much an outcome violated current value beliefs about the chosen option. Lower-than-expected choice outcomes resulted in stronger pupil constriction compared to higher-than-expected ones, a response pattern consistent with value coding [55]. As the reward prediction error term multiplied with the learning rate updates the value of the chosen stimulus, reward prediction error responses in the pupil after feedback are consistent with our finding that chosen value uniquely modulated pupil dilation prior to a choice. We can only speculate about the similarity to the reward prediction error firing pattern of phasic dopamine neurons [56–59] that briefly activate after higher-than-expected outcomes and deactivate after lower-than-expected outcomes.

Alternatively, the observed correlation between late feedback-related pupil constriction and signed reward prediction errors could be driven by differences in saliency between unexpected positive and negative outcomes. It has been shown that contrast-based stimulus saliency modulates the magnitude of transient pupil responses, with more salient stimuli evoking a larger peak-to-peak (i.e. larger dilation and constriction) pupil response [60, 61]. While we controlled for the physical stimulus saliency in our experiment, some experimental events could have been more salient than others. Due to reinforcement learning, unexpected negative outcomes occurred less often than unexpected positive outcomes, which might have rendered them subjectively more salient events. Thus, increased subjective saliency could explain the stronger feedback-related pupil constriction observed after unexpected negative events.

We found that pupil responses systematically tracked key components of reinforcement learning, however, important differences were observed with a later recall phase. Only during learning, but not during transfer, was pupil dilation prior to choice modulated by choice value; a difference that may indicate different underlying cognitive processes that drive value-based choices [62, 63]. Why was this the case, in a task where participants had to make value-based decisions in both experimental phases? One important difference between the learning and transfer phase was the presentation of choice feedback, thus, the ability to learn from choice outcomes. In the learning phase, only three stimulus pairs were presented and each choice was followed by feedback to allow learning. In the transfer phase, our objective was to assess already internalized choice value by confronting participants with novel stimulus pair combinations of previously acquired reinforcement values. Hence, in this phase, choices were not followed by feedback, and choice value representations could not be changed.

Dopamine, particularly in the striatum [64], plays an important role during reinforcement learning [65]. Striatal dopamine strengthens actions that lead to rewarding outcomes and weakens those that lead to aversive ones [22, 32, 36]. It thereby flexibly adapts behavior to maximize future reward. In the transfer phase, value beliefs are consolidated and dopamine no longer plays an important role in learning or modulating choice behavior. Information used to make a value-based choice can be retrieved from memory, guided by structures that encode learned value representations, such as the ventromedial prefrontal cortex [63, 66]. Our finding that pupil dilation signaled upcoming choice value only during learning, could mean that the pupil is particularly sensitive to contingency learning [67], thus, to learning the mapping between actions and outcomes.

Whether, and in what way, dopamine modulates the pupil response has yet to be determined, but several lines of research show promising results [7, 20, 21, 68, 69]. It is likely that such modulations occur via interactions with the noradrenergic locus coeruleus (LC), a brainstem nucleus that is often linked to pupil dilation in micro-stimulation and decision-making studies [68, 70–73]. As these studies also found that activity in other brain areas correlated with pupil responses [60, 61, 70, 71], including the dopaminergic ventral tegmental area [68], this further suggests multiple (interacting) sources driving pupil size. In support of interactions between the noradrenergic and dopaminergic system in modulating pupil size, the LC and dopaminergic midbrain structures have dense reciprocal connections and receive a common top-down projection from the prefrontal cortex [74]. Moreover, both systems play important, yet different, roles in reward-learning and motivational behavior [73, 75], which suggest they both might play important roles in modulating the pupil during reinforcement learning.

While we observed the pupil to systematically track key components of reinforcement learning, could these results alternatively be explained by effects of attention? The pupil has long been used as an index of attention, as the amount of attention paid to a stimulus determines the amount of transient pupil dilation [76–79]. In our study, when choices where made, attention was most likely shifted toward the chosen option, which could have driven phasic pupil dilation during choice. While this explanation fits our finding in the learning phase, where pre-choice pupil dilation scaled with the value of the to-be chosen option, it does not fit with the absence of this relation in the transfer phase, even though participants paid attention to the chosen options, as they performed the task well. Neither does attention -and its relation to phasic pupil dilation- explain why feedback-related pupil constriction in the learning phase scaled with how much an outcome violated choice value beliefs. Thus, while attention likely plays an important role in value-based decision-making [42, 80–82], the observed patterns of results here cannot be solely explained by effects of attention.

To conclude, our study provides evidence that the pupil is a reliable indicator of value-based decision-making during active reinforcement learning. Pupil responses signaled the processing of value up to a choice and the subsequent evaluation of choice outcomes in terms of uncertainty and violations of value beliefs. There were several aspects to our approach that enabled us to establish these specific relations and to move beyond previous work linking the pupil to reward. First, our computational approach enabled us to characterize the full temporal profile of value-based decisions in the pupil, thereby relating different decision and evaluation processes to different components of the pupil response. These relationships could only be obtained using a ridge regression deconvolution approach that enabled us to disentangle the multiple underlying cognitive processes that impacted pupil size within a single regression analysis. Second, these specific relations could only be established with the use of classical learning theories that provided us access to participants’ developing value beliefs, and the underlying choice considerations thought to support value-driven decisions. This also highlights our use of internal, or subjective, value estimates to relate to the pupil. This contrasts previous studies that used externally derived value estimates to investigate reward-related effects on pupil size [8, 12, 20, 21, 69]. Lastly, our study describes the temporal evolution of reinforcement learning in the pupil, thereby providing evidence that the pupil can be used to non-invasively track the reinforcement learning process as it takes place. Future studies that combine functional brain imaging and pupillometry will have to further specify the brain areas that contribute to the value-based pupil response.

## Materials and methods

### Ethics statement

The Ethics Committee of the Vrije Universiteit Amsterdam approved this study.

### Participants

Forty-two healthy participants with normal to corrected to normal vision completed the experiment (10 males; mean age = 24.9; age range = 18-34 years). They were paid 16€ for 2 hours of participation and earned an additional performance bonus (mean = 10.2€, SD = 1.8). The ethical committee of the Vrije Universiteit Amsterdam approved the study and written informed consent was obtained from all participants. Eight participants were excluded from analyses due to the following reasons: inadequate fixation to the center of the screen (N = 4), reporting more than three unique stimulus pairs in the learning phase (N = 1) and (almost) perfect choice accuracy in the learning phase, which complicated behavioral model fitting (N = 3), resulting in a total of 34 participants for the analyses.

### Task & procedure

Participants were seated in a dimly lit, silent room with their head positioned on a chin rest, 60 centimeters away from the computer screen. They received written information about the general purpose of the experiment, after which they completed a 30-trial practice session of the learning phase. Subsequently, participants completed for the learning phase 6 runs of 60 trials each (360 trials in total, 120 presentations of each stimulus pair), with small breaks in-between runs. After each run, the earned number of points was displayed. At the end of the learning phase, the total number of earned points was converted into a monetary bonus. Directly after the learning phase, participants entered the transfer phase. They completed 5 runs of 60 trials each (300 trials in total, 20 presentations per stimulus pair), with small breaks in-between runs. Overall choice accuracy was displayed at the end of the transfer phase.

### Stimuli & trial structure

Stimuli were presented on a 21-inch Iiyama Vision Master 505 MS103DT with a spatial resolution of 1024 x 768 pixels, at a refresh rate of 120Hz, with mean luminance 60 cd/m^2^. Experiments were programmed in OpenSesame and data analysis were performed using custom software written in Python, using Numpy (v1.11.2), Scipy (v0.18.1), FIRDeconvolution (v0.1.dev1), Hedfpy (v0.0.dev1), MNE (v0.14) and PyStan (v2.14) packages. Luminance effects on pupil size were minimized by keeping the background luminance of the display constant. Color stimuli were near-isoluminant to each other and the background (set via a flicker-fusion color calibration test carried out once at the start of the experiment). To account for further luminance bias effects, each participant had a unique color pair (red-blue; yellow-dark blue; green-magenta) to reward probability mapping (AB, CD, EF) that was counterbalanced in order (e.g. red-blue or blue-red for AB).

In each learning phase trial, participants continuously fixated on a central white fixation dot. After 500ms (SD = 200ms), two colored stimuli (1.26°x1.26° visual angle) appeared at the horizontal meridian left and right from the central fixation dot at a distance of 5.04° visual angle. Participants made a choice for one of the options using the ‘K’ (left choice) and ‘L’ (right choice) keys. A choice was highlighted by a small dark gray arrow (150ms) pointing in the direction of the chosen option. After a random interval drawn from a Gaussian distribution with a mean of 1500ms (SD = 300ms), the choice was followed by auditory feedback, indicating reward (+0.1 points; 500ms “correct” sound) or no reward (500ms; pure sine tone at 300Hz). Omissions or response times (RTs) longer than 3500ms were followed by a neutral tone (500ms; pure sine tone at 660Hz). Inter-trial intervals were drawn from a Gaussian distribution with a mean of 3000ms (SD = 300ms) Trials of the transfer phase followed the same trial structure as trials in the learning phase, but had a shorter duration as choices were not followed by feedback.

### Behavioral analysis

Choices and RTs were recorded for all trials in the learning and transfer phase. RT on every trial was computed as the time from onset of the stimulus pair until the choice (key press). Trials with RTs below 150ms or above the RT deadline of 3500ms were removed from all analyses. As a choice between two options in the learning phase was never necessarily “correct”, we defined the selection of the optimal option (more reinforcing option of the presented pair) as a correct choice. For the transfer phase, value conflict on a particular trial was defined on the basis of the experimental reinforcement value difference between the presented stimuli, where smaller value differences were associated with higher conflict.

### Computational model

Choices during the learning phase were fit with a reinforcement learning (“Q-learning”) model [23, 83]. The Q-learning model has an extensive theoretical background in which decision-making is explicitly evaluated [84, 85] and has been successfully applied in a range of domains. Examples of this are genetics [32, 86], clinical settings [54, 63, 87–89], attention [24], decision bias [34] and risk [38]. For each option, the model estimates its expected value, or “Q-value”, on the basis of individual sequences of choices and outcomes. All Q-values were set to 0.5 before learning. After each choice, the chosen option’s Q-value is updated by learning from feedback that resulted in an unexpected outcome, which is captured by the RPE, *r*_i_(*t*) − *Q*_i_(*t*). Thus, the Q-value for option *i* on the next trial *t* is updated depending on the outcome, *r*, using the following formula:

$$\begin{array}{c} Q_{i}\left(t+1\right)=Q_{i}\left(t\right)+\{\begin{array}{cc} \alpha_{Gain}\left[\;r_{i}\left(t\right)-Q_{i}\left(t\right)\right]\; & \text{if}\;r\;=1\\ \alpha_{Loss}\left[\;r_{i}\left(t\right)-Q_{i}\left(t\right)\right]\; & \text{if}\;r\;=0 \end{array} \end{array}$$ (1)

where parameters 0 ≤ *α*_Gain_, *α*_Loss_ ≤ 1 represent positive and negative learning rates, respectively, that determine the magnitude by which value beliefs are updated depending on the RPE. We modeled separate learning rates, as different striatal subpopulations are involved in positive and negative feedback learning [35–37, 90] and individuals tend to learn more from positive feedback [24, 25, 34]. Modeling two learning rates was further validated by comparing this hierarchical Q-learning model to a hierarchical Q-learning model with only one learning rate. Model selection was based on individual AIC and BIC values and supported the use of two learning rates, as indicated by lower AIC and BIC values (mean AIC 1*α* = 234, mean AIC 2*α*’s = 218; mean BIC 1*α* = 242, mean BIC 2*α*’s = 230). Given the Q-values, the probability of selecting one option over the other (e.g. selecting option A over B) was described by a softmax choice rule:

$$\begin{array}{c} P_{A}\left(t\right)=\frac{exp(\beta \cdot Q_{A}\left(t\right))}{exp\left(\beta \cdot Q_{B}\left(t\right)\right)+exp\left(\beta \cdot Q_{A}\left(t\right)\right)} \end{array}$$ (2)

Here, 0 ≤ *β* ≤ 100, or the explore-exploit parameter, described the sensitivity to option value differences, where larger *β* values indicates greater sensitivity, and more exploitative choices, for options with relative higher reward values.

### Bayesian hierarchical modeling procedure

The Q-learning model was fit using a Bayesian hierarchical fitting procedure, where individual parameter estimates were drawn from group-level parameter distributions that constrained the range of possible individual parameter estimates. This procedure allowed for the simultaneous estimation of group-level and individual-level parameters [26, 91], thereby capitalizing on the statistical strength offered by the degree to which participants are similar with respect to the model parameters as well as taking into account individual differences [92].

As shown in Fig 1*C*, our model was implemented following [24, 25]. Variables *r*_i_(*t*-1) (outcome for participant *i* on trial *t*-1) and *ch*_i_(*t*) (choice of participant *i* on trial *t*) were obtained from the behavioural data. Per-participant parameter estimates *α*_*Gi*_ (*α*_*Gain*_ participant *i*), *α*_*Li*_ (*α*_*Loss*_ participant *i*) and *β*_*i*_ (*β* participant *i*) were modeled using a probit transformation *z*’_i_ ($\alpha_{Gi}^{\prime }$, $\alpha_{Li}^{\prime }$, $\beta_{i}^{\prime }$). The probit transformation is the inverse cumulative distribution function of the normal distribution that can be used to specify a binary response model. *z*’_i_ were drawn from group-level normal distributions with mean *μ*_*z*′_ and standard deviation *δ*_*z*′_. A normal prior was assigned to group-level means $\mu \;{\text{z}}^{’}∼N(1,\;0)$ and a uniform prior to the group-level standard deviations $\delta \;{\text{z}}^{’}∼U(1,\;1.5)$ [26]. The Bayesian hierarchical model was implemented in STAN [93] and fit to all trials of the learning phase that fell within the correct response time window 150ms ≤ RT ≤ 3500ms, (mean = 99.5% of trials, SD = 0.8%). Multiple chains were generated to ensure convergence, which was evaluated by the Rhat statistic [94]. The Rhat statistic confirmed convergence of the fitting procedure (i.e., all Rhats were equal to 1.0). We also tested whether the derived per-participant parameters could simulate choices that were qualitatively similar to the observed choices originally used for fitting. Here, choices were simulated 100 times for each participant using the mode of the derived per-participant parameter distribution. Simulated choice accuracy was averaged across simulations and evaluated against the observed choice data (Fig 2*C*).

### Quantifying single-trial estimates

The modes of the per-participant posterior parameter distributions were selected to describe individual positive and negative learning rates (*α*_Gain_, *α*_Loss_) and relative reward sensitivity (*β*). In the learning phase, these per-participant parameter estimates were used to quantify Q-values and RPEs on each trial. Specifically, we quantified for each trial the value of the option that was chosen and the alternative unchosen option. In the transfer phase, when participants did not receive feedback about their choices, we investigated how previously learned value related to pupil responses during value-based decisions. To do so, we selected the final Q-value estimates for each option (i.e. at the end of the learning phase) and used these values to quantify for each trial the value of the chosen and unchosen stimulus, given the individual sequences of choices. All obtained single-trial variables were used as covariate regressors in a deconvolution analysis (described below), to investigate how they dynamically varied with trial-by-trial fluctuations in transient pupil responses in the learning and transfer phase.

### Pupillometry: Preprocessing

The diameter of the pupil was recorded at a 1000Hz using an EyeLink 1000 Tower Mount (SR Research). The eye-tracker was calibrated prior to each run. Blinks and saccades were detected using standard EyeLink software with default settings and Hedfpy, a Python package for preprocessing eye-tracking data. Periods of data loss during blinks were removed by linear interpolation, using an interpolation time window of 200ms before until 200ms after a blink. Blinks not identified by the manufacturer’s software were removed by linear interpolation around peaks in the rate of change of pupil size, using the same interpolation time window. The interpolated pupil signal was band-pass filtered between 0.05Hz and 4Hz, using third-order Butterworth filters, z-scored per run, and resampled to 20Hz. As blinks and saccades have strong and relatively long-lasting effects on transient pupil size [40, 95], these influences were removed from the data, as follows. Blink and saccade regressors were created by convolving all blink and saccade events with their standard Impulse Response Function (IRF) [40, 96, 97]. These convolved regressors were used to estimate their responses in a General Linear Model (GLM), after which we used the residuals of this GLM for further analysis. For the subsequent deconvolution analysis, trials were removed in which participants made a saccade towards either of the two presented colored stimuli (i.e. saccades exceeding 3.3° visual angle away from fixation) to ensure that pupil responses were not affected by eye movements (percentage removed trials, mean = 4.8%; SD = 4.5%; range = 0.0%-16.3%).

### Pupillometry: Deconvolution analysis

#### Learning phase

Transient pupil responses were analyzed using FIRDeconvolution, a Python package used to perform Finite Impulse Response fits [39]. For the analysis of the learning phase, a design matrix was constructed that estimated pupil time courses of the following 3 transient event types: the onset of the choice options (start of the decision interval), choice (keypress) and feedback (auditory tone). Time courses of the onset of the options and feedback were estimated in the interval -0.5s. pre-event until 3.0s. post-event. The time course of the choice was estimated in the interval -2.0s. pre-event until 1.5s post-event, as decision-related pupil dilation is predominantly driven prior to the behavioral report [5, 43]. Further, the sustained drive of pupil size during the decision interval, defined as the time period from onset of the options until the choice, was estimated by a boxcar regressor. The boxcar regressor expanded each trial’s RT (in samples) and was normalized by dividing the height of the boxcar by the mean RT of the regressor. This procedure ensured that the estimated IRF of all transient and sustained regressor types were comparable. Lastly, the design matrix included 2 stick regressors to estimate the pupil time course of the following nuisance events: the onset of the fixation dot and the offset of the options from the screen. Pupil time courses of both events were estimated -0.5s. pre-event until 3.0s. post-event. No intercept was added to the design matrix as ridge regression (described below) requires centered dependent and independent variables [98]. For the decision interval, we investigated how value beliefs about presented options affected pupil size by adding single-trial chosen and unchosen Q-value estimates as covariates to the design matrix. The resulting regression coefficients -describing the time course of the correlation between chosen and unchosen value and pupil size- were subtracted from each other to quantify the correlation of the Q-value difference (ΔValue) and pupil size. For the feedback interval, the same Q-value estimates were used as in the decision interval. This allowed us to investigate how value beliefs about a recent choice affected pupil size after receiving choice feedback. Thus, by using the same Q-value estimates for choice and feedback intervals, we were able to investigate the effects of value predictions (choice) and value evaluations (feedback) on pupil size. Finally, we added single-trial RPE estimates to the design matrix to investigate how violations of choice beliefs affected the feedback-related response. All covariate regressors were z-scored per participant, to ensure unbiased across-subject comparisons of deconvolution beta weights.

#### Transfer phase

The design matrix for the deconvolution analysis of the transfer phase was identical to that of the learning phase, with two exceptions: (1) the pupil time course for feedback events was not estimated, as no feedback events occurred during this phase, (2) a stick regressor was included to investigate the effects of choice conflict on pupil responses. Choice conflict was determined the basis of experimental reinforcement value differences between the presented options, where trials were divided into three bins (10-20%; 30-40% and 50-60%), that corresponded to large, medium and small choice conflict between options.

### Pupillometry: Ridge regression

We implemented the deconvolution analysis using cross-validated ridge regression, which allows one to find the general solution to a least-squares problem that would be unstable due to multicollinearity of regressors [98]. Ridge regression penalizes, or shrinks, regression coefficient weights towards zero to reduce the estimation variance on the coefficients:

$$\begin{array}{c} {\hat{\beta }}_{ridge}={\left(X^{T}X+\lambda I\right)}^{-1}X^{T}y \end{array}$$ (3)

Here, *y* is the pupil time series signal and *X* is the design matrix consisting of a set of vectors that contain ones at all sample times relative to the event timings of which we estimated the pupil response, and zeros elsewhere. The identity matrix, *I*, is multiplied by λ ≥ 0, a tuning parameter that controls the strength of the penalty term. If λ = 0, the linear regression solution is obtained, λ = ∞, ${\hat{\beta }}_{ridge}=0$. To obtain for each participant the optimal λ value, we applied cross validation on the pupil time series data. Here, the pupil data was divided into a training and test set. A weight matrix was obtained for each λ value (range = 0 ≤ λ ≤ 1), using the training set, and was used to predict the test set. This process was repeated for 20 different selections of training and test sets, and the best λ value was selected based on its prediction accuracy. The resulting regression, ${\hat{\beta }}_{ridge}$, contained the deconvolved pupil responses of all separate event types.

### Statistical comparisons

Nonparametric cluster-based permutation *t*-tests [99–101] were used to test for significant regression coefficients and to correct for multiple comparisons over time. Briefly, for each time point of a time series signal, *t*-tests were performed on each set of across-subject coefficient values. The cluster size was determined by the number of contiguous timepoints for which the *t*-test resulted in *P*<.05. The observed cluster size was then compared to a random permutation distribution of maximal cluster sizes: the proportion of random clusters resulting in a larger size than the observed one determined the *P*-value, corrected for multiple comparisons.

To assess the effects of chosen and unchosen value covariates on pupil size across the decision interval, we summed each regressor’s coefficient values locked to the start (option onset) and locked to the end of the decision interval (the moment of choice), while discarding their post-choice effects. We normalized the summed regressor coefficient values by the number of samples they explained of the pupil time series signal. The resulting averaged, normalized regressor coefficient values were used in a repeated measures ANOVA to test for main and interaction effects on pupil size, both for the learning and transfer phase.

Across-subject analyses of the relation between pupil responses and computational model parameters were calculated using bootstraps [102]. We randomly drew with replacement 10,000 new pupil size—model parameter estimate pairs which were used in the across-subject GLM. From the resulting bootstrapped regression coefficients, 68% confidence intervals were calculated using a percentile approach. *P*-values calculations were based on a two-sided hypothesis test, with the *P*-value being the fraction of the bootstrap distribution that fell below (or above) 0.

## Supporting information

S1 Fig Pupil responses predict individual differences in value-based decision-making. We performed an across-subjects GLM describing the relation between pupil responses and estimated model parameters across time. Average choice-locked pupil dilation (A) at the time of the behavioral report (*t* = 0) was uniquely predicted by individual differences in relative reward sensitivity (*β*-parameter; B). (C): Average feedback-related pupil dilation (1s. post-event) and pupil constriction (2s. post-event) were uniquely predicted by individual differences in positive learning rate (*α*_*Gain*_-parameter; D). (E,F): Average choice-locked pupil dilation in the learning and transfer phase related in a highly similar fashion to the derived model parameters (compare panels B and F), suggesting these underlying mechanisms affected choice-locked pupil dilation in a similar way. Lines (*dashed & solid*) and shaded error bars of represent mean ± s.e.m of across-subject modulations (N = 34). Horizontal significance designators indicate time points where regression coefficients significantly differentiate from zero (*P*<.05), based on cluster-based permutation tests (n = 1000).
(TIF)

S2 Fig Differences in tonic pupil size do not explain observed differences in pre-choice pupil size modulation between the learning and transfer phase. Chosen value modulated choice-locked pupil dilation prior to choice in the learning, but not in the transfer phase. Could this differential pupil size modulation be driven by slow fluctuations in tonic pupil size, that are known to affect the magnitude of concurrent phasic pupil responses? We tested this hypothesis using the unfiltered pupil time series data from the learning and transfer phase. As these data contain all temporal frequencies in the pupil size signal, they provide a clear view on potential tonic pupil size differences between the learning and transfer phase. Before making any comparisons, we first calculated each participant’s average pupil size across the entire experiment and subtracted this value from the blink-interpolated pupil time series data. This procedure corrected for inter-individual differences in raw baseline pupil diameter that can be caused by several confounding factors such as differences in ambient lightning or age [40, 103], improving power for the comparison of the different conditions. Next, for each subject the blink-interpolated pupil data of the learning and transfer phase was divided in trial epochs and mean pupil size per trial was calculated—this effectively constitutes a low-pass filtering operation and is standard in the literature [4, 104]. (A), Averaged across subjects (N = 34), no systematic differences in tonic pupil size were observed between the learning and transfer phase. This finding speaks against the hypothesis that differences in tonic pupil size explain the observed differential modulation of pre-choice pupil dilation between the learning and transfer phase. However, the observed slow fluctuations of tonic pupil size followed a consistent pattern that was characterized by high tonic pupil size at the start of each run (consisting of 60 trials) and that progressively decreased until the end of a run; a pattern that has been reported before [40, 105] and is thought to reflect the evolution of general vigilance during each experimental run. (B) Box plots representing tonic pupil size averaged across all trials of all participants (N = 34) of the learning and transfer phase largely overlap, showing that tonic pupil size does not systematically differ between the learning and transfer phase. Shaded error bars represent bootstrapped 95% confidence intervals of the mean (n = 1000).
(TIF)

S3 Fig Surprise does not modulate feedback-related pupil dilation. We asked whether feedback-related pupil dilations scaled with the unexpectedness or the uncertainty of choice outcomes. We quantified how value beliefs about a recent choice modulated dilation after receiving positive versus negative feedback. Receiving positive (A, *black line*) versus negative (B, *black line*) feedback resulted in equally strong feedback-related pupil dilation at time of maximal dilation (cluster *P* = .23), indicating that feedback valence did not drive pupil dilation. If these feedback-related pupil dilations were modulated by surprise, unexpected feedback with respect to current value beliefs should increase dilation. That is, for positive feedback the difference in value beliefs (chosen—unchosen) should correlate negatively with pupil dilation whereas for negative feedback it should correlate positively. We found that the correlation between value beliefs and pupil dilation was highly similar in response to both positive and negative feedback (C). This pattern of results indicates that uncertainty rather than surprise drives feedback-related pupil dilations. Statistics based on cluster-based permutation tests (n = 1000).
(TIF)
