# Correction: How pupil responses track value-based decision-making during and after reinforcement learning

**DOI:** 10.1371/journal.pcbi.1007031

**Published:** 2019-05-06

**Authors:** Joanne C. Van Slooten, Sara Jahfari, Tomas Knapen, Jan Theeuwes

The caption of Fig 5 is incorrect. The authors have provided a corrected version here.

**Fig 5:**
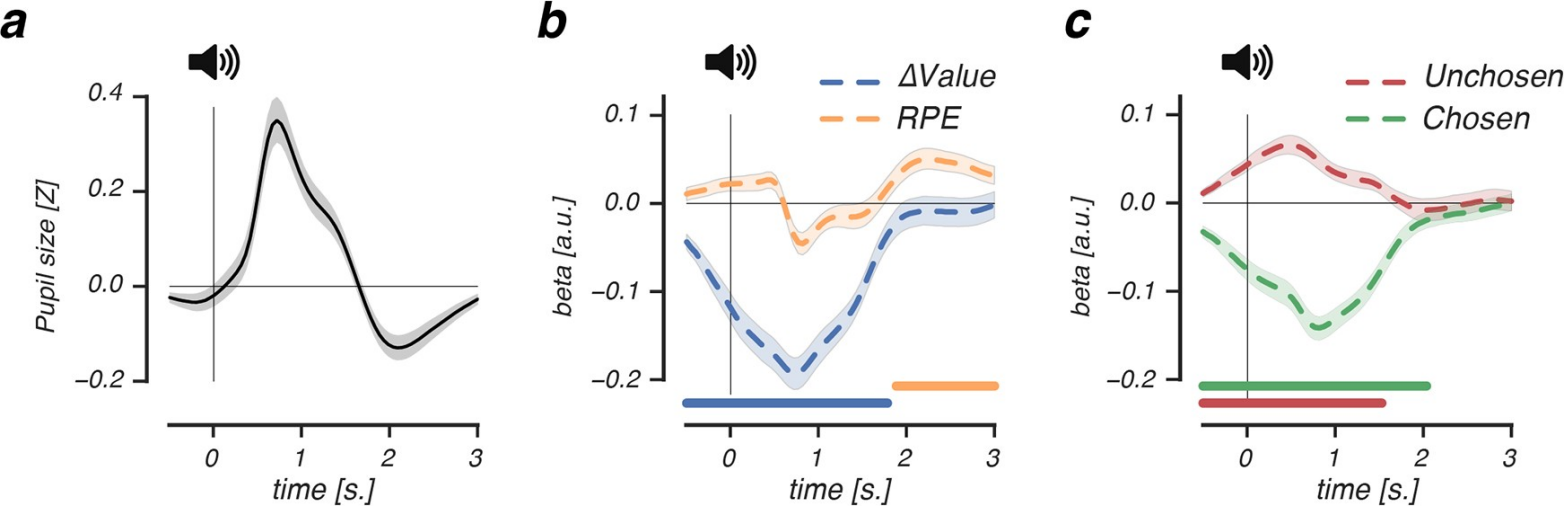
The feedback-related pupil response reflects value uncertainty and reward prediction errors. (A-C), Beta coefficients accounting for the feedback-related pupil response in the learning phase. (A) The feedback-related pupil response was characterized by early dilation (1s. post-event) and late constriction (2s. post-event). (B), Early in time (1s. post-event), feedback-related pupil dilation correlated negatively with the learned value difference of recently presented options (ΔValue, blue dashed line). Late in time (2s. post-event), feedback-related pupil constriction correlated positively with signed RPEs (orange dashed line). (C), Both low value beliefs about the recently chosen option and high value beliefs about the unchosen option increased feedback-related pupil dilation, already prior to the moment of feedback (at t = 0). Lines and shaded error bars represent mean ± s.e.m. of within-subject modulations. Horizontal significance designators indicate time points where regression coefficients significantly differentiate from zero (P < .05). Statistics based on cluster-based permutation tests, n = 1000.
