# Supplementary figures and images for: How pupil responses track value-based decision-making during and after reinforcement learning
Source: PLoS Comput Biol. 2018 Nov 30;14(11):e1006632. doi: 10.1371/journal.pcbi.1006632 (PMC6291167; doi:10.1371/journal.pcbi.1006632)

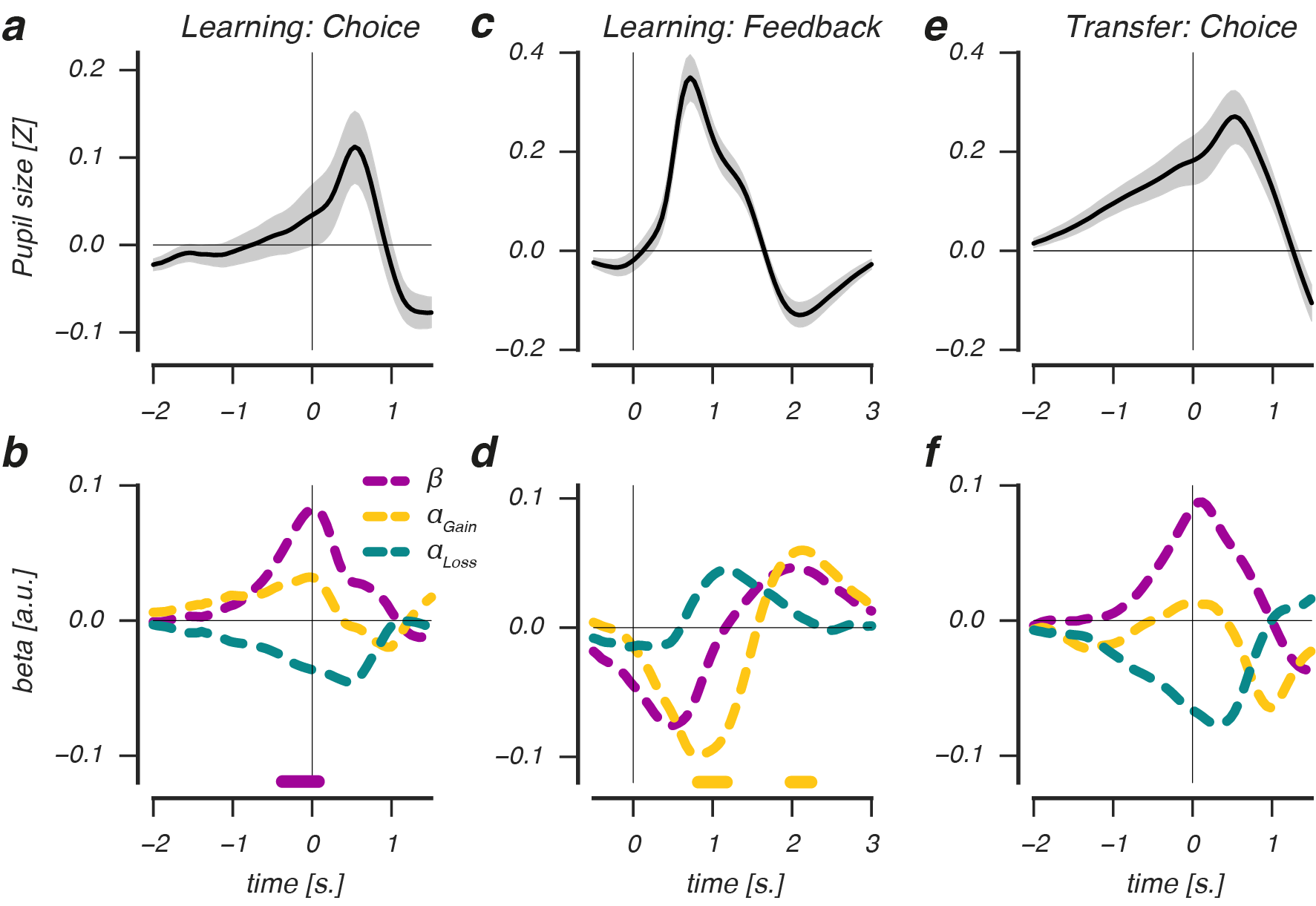

Supplement: S1 Fig — We performed an across-subjects GLM describing the relation between pupil responses and estimated model parameters across time. Average choice-locked pupil dilation (A) at the time of the behavioral report (t = 0) was uniquely predicted by individual differences in relative reward sensitivity (β-parameter; B). (C): Average feedback-related pupil dilation (1s. post-event) and pupil constriction (2s. post-event) were uniquely predicted by individual differences in positive learning rate (αGain-parameter; D). (E,F): Average choice-locked pupil dilation in the learning and transfer phase related in a highly similar fashion to the derived model parameters (compare panels B and F), suggesting these underlying mechanisms affected choice-locked pupil dilation in a similar way. Lines (dashed & solid) and shaded error bars of represent mean ± s.e.m of across-subject modulations (N = 34). Horizontal significance designators indicate time points where regression coefficients significantly differentiate from zero (P<.05), based on cluster-based permutation tests (n = 1000). (TIF) [file pcbi.1006632.s001.tif]

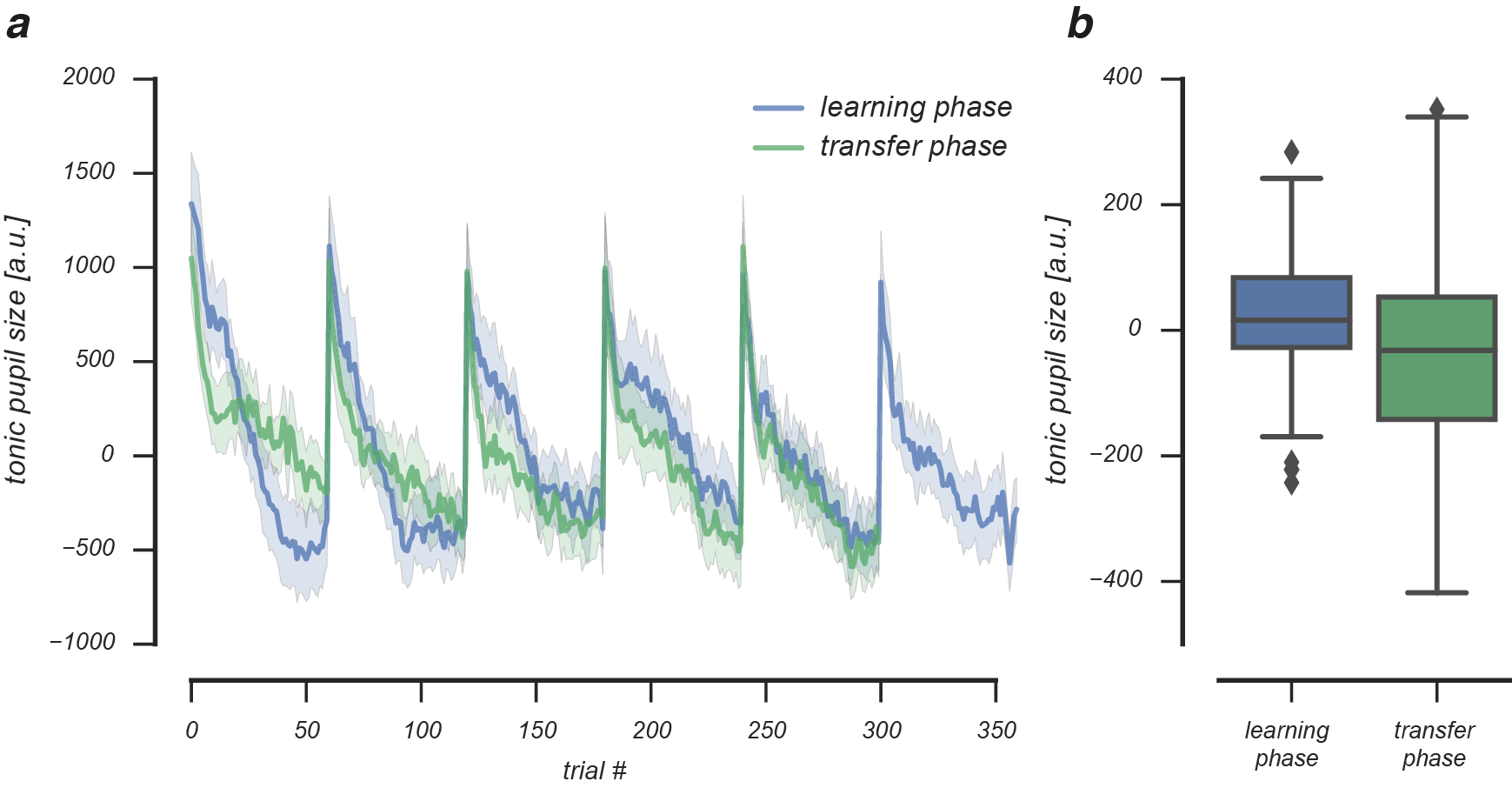

Supplement: S2 Fig — Chosen value modulated choice-locked pupil dilation prior to choice in the learning, but not in the transfer phase. Could this differential pupil size modulation be driven by slow fluctuations in tonic pupil size, that are known to affect the magnitude of concurrent phasic pupil responses? We tested this hypothesis using the unfiltered pupil time series data from the learning and transfer phase. As these data contain all temporal frequencies in the pupil size signal, they provide a clear view on potential tonic pupil size differences between the learning and transfer phase. Before making any comparisons, we first calculated each participant’s average pupil size across the entire experiment and subtracted this value from the blink-interpolated pupil time series data. This procedure corrected for inter-individual differences in raw baseline pupil diameter that can be caused by several confounding factors such as differences in ambient lightning or age [40, 103], improving power for the comparison of the different conditions. Next, for each subject the blink-interpolated pupil data of the learning and transfer phase was divided in trial epochs and mean pupil size per trial was calculated—this effectively constitutes a low-pass filtering operation and is standard in the literature [4, 104]. (A), Averaged across subjects (N = 34), no systematic differences in tonic pupil size were observed between the learning and transfer phase. This finding speaks against the hypothesis that differences in tonic pupil size explain the observed differential modulation of pre-choice pupil dilation between the learning and transfer phase. However, the observed slow fluctuations of tonic pupil size followed a consistent pattern that was characterized by high tonic pupil size at the start of each run (consisting of 60 trials) and that progressively decreased until the end of a run; a pattern that has been reported before [40, 105] and is thought to reflect the evolution of general vigilance [file pcbi.1006632.s002.tif]

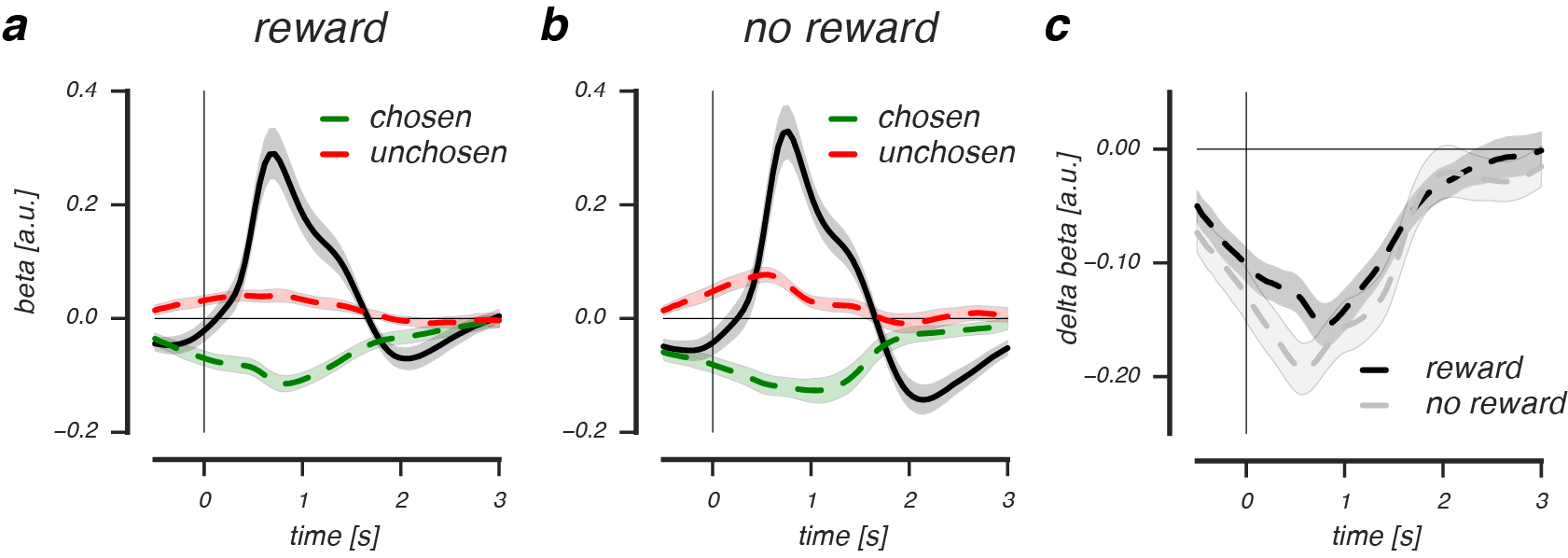

Supplement: S3 Fig — We asked whether feedback-related pupil dilations scaled with the unexpectedness or the uncertainty of choice outcomes. We quantified how value beliefs about a recent choice modulated dilation after receiving positive versus negative feedback. Receiving positive (A, black line) versus negative (B, black line) feedback resulted in equally strong feedback-related pupil dilation at time of maximal dilation (cluster P = .23), indicating that feedback valence did not drive pupil dilation. If these feedback-related pupil dilations were modulated by surprise, unexpected feedback with respect to current value beliefs should increase dilation. That is, for positive feedback the difference in value beliefs (chosen—unchosen) should correlate negatively with pupil dilation whereas for negative feedback it should correlate positively. We found that the correlation between value beliefs and pupil dilation was highly similar in response to both positive and negative feedback (C). This pattern of results indicates that uncertainty rather than surprise drives feedback-related pupil dilations. Statistics based on cluster-based permutation tests (n = 1000). (TIF) [file pcbi.1006632.s003.tif]
